# Regulation of the growth performance and the gastrointestinal microbiota community by the addition of defective pear fermentation to feed of small-tailed Han sheep

**DOI:** 10.3389/fmicb.2024.1358033

**Published:** 2024-04-04

**Authors:** Hongxin Peng, Pinpin Chen, Rui Guo, Zheng Zhou, Yafei Chen, Ping Xu, Huawei Su, Yuncai Xiao, Hui Jiang

**Affiliations:** ^1^Key Laboratory of Tarim University Husbandry Science and Technology, College of Animal Science and Technology, Tarim University, Alar, China; ^2^National Key Laboratory of Agricultural Microbiology, Huazhong Agricultural University, Wuhan, China; ^3^State Key Laboratory of Animal Nutrition, College of Animal Science and Technology, China Agricultural University, Beijing, China

**Keywords:** defective pear fermentation, small-tailed Han sheep, growth performance, serum antioxidant, gastrointestinal microbiota community

## Abstract

This study investigated the effects of defective pear fermentation (DPF) diets on growth performance and gastrointestinal microbial communities in 60 healthy male small-tailed Han sheep, aged 90 days. The sheep were randomly divided into four groups, each consisting of three replicates with five sheep per replicate. Initially, all groups received a basal diet for seven days during the adaptation stage. Subsequently, for 60 days, group C (control) was fed a basal diet, group X received a basal diet with 2% DPF, group Y had a basal diet with 4% DPF, and group Z was fed a basal diet with 6% DPF. The results indicated that group Y experienced a significant increase in average daily gain (ADG) and average daily feed intake (ADFI). The addition of DPF significantly elevated the levels of GSH-Px and notably reduced MDA content compared to group C. Analysis of gastrointestinal microbiota showed that groups receiving DPF had increased relative abundances of *Lachnospiraceae_NK3A20_group, norank_f p-2534-18B5_gut_group, Acetitomaculum, Actinobacteriota, Bacteroidota* and *Ruminococcus_gauvreauii_group*, and decreased abundances of *Proteobacteria, Prevotella, Staphylococcus*, and *Psychrobacter* compared to group C. Group X exhibited the highest relative abundance of *Olsenella*, while group Y showed a significant increase in *unclassified_f Lachnospiraceae* compared to the other groups. Bacterial function prediction indicated that pathways related to energy metabolism were more prevalent in group X and Y. This study preliminarily confirms the feasibility of using DPF as feed additives, providing a foundation for further research and evaluation of DPF's application in animal production.

## Graphical Abstract

The schematic study design of the animal experiment. 
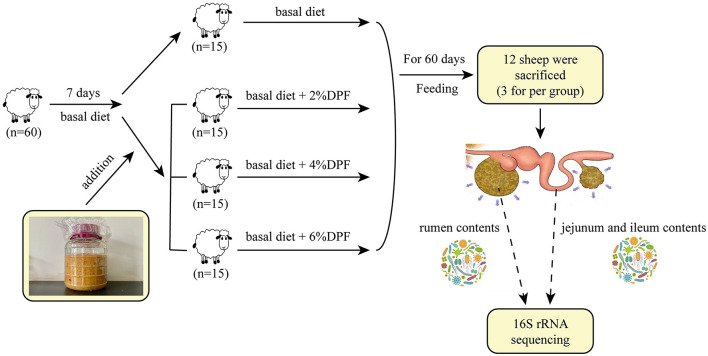

## 1 Introduction

Xinjiang Uygur Autonomous Region is a prominent province for animal husbandry in China, with residents primarily consuming beef and mutton (Wang C. et al., 2023). With China's rapidly growing economy, the demand for meat products is increasing (Dawut and Tian, 2021). However, the development of animal husbandry in Xinjiang is heavily reliant on high-quality forage resources, a crucial factor that challenges the industry's growth (Thornton and Gerber, 2010; Godber and Wall, 2014).

In recent years, the cultivation of fragrant pears has become a significant sector in Xinjiang's forestry and fruit industry (Niu et al., 2019; Wang Z. et al., 2023). By the end of 2022, Bazhou's fragrant pear planting area reached 489,600 mu, yielding a total output of 386,400 tons (Ma et al., 2023). During harvesting, transportation, and marketing, a portion of the fruits, approximately 30% of total production, are deemed commercially valueless due to damage or spoilage. These are known as residual fruits and their abundance, coupled with their susceptibility to natural deterioration, poses environmental pollution risks and resource wastage (Guo, 2022).

Following China's 2020 ban on antibiotic utilization, there has been an increase in research on alternative feed additives, such as Chinese herbal additives, micro-ecological preparations, and plant essential oil extracts (Meena et al., 2022; Yang et al., 2022; Caroprese et al., 2023; Guo et al., 2023; Kiernan et al., 2023; Ramdani et al., 2023; Varga-Visi et al., 2023). While most research has focused on swine and poultry, applications in sheep are less common. At present, most of the research is to replace the traditional feed with unconventional feed to explore its effects on the growth performance and gastrointestinal function of animals. However, there are few studies on improving animal growth performance by adding fruit by-product or fermentation. The following are several studies on the use of fruit by-products in animals: Cheng (2021)'s study found that adding 8% grape pomace increased the daily feed intake of Tan sheep. Ao et al. (2022) study showed that adding 10% fermented apple pomace to the feed of weaned piglets could increase their average daily gain. As report goes, the antioxidant activity in plasma of lambs was increased after feeding 10% fermented apple pomace diet (Rodríguez-Muela et al., 2015). These few studies have revealed that fruit by-product or fermentation has a positive effect on animal growth, which is one of the reasons why our team chose to use defective pear fermentation as an additive.

In these contexts, we chose small-tail Han sheep as our study subject, one of China's superior hybrid breeds which is known for its rough feeding resistance, strong adaptability, high fecundity, and rapid growth rate (Lu et al., 2005; Jing et al., 2022). To enhance the preservation and utilization of defective pear resources in animal husbandry, this study utilized defective pear as a carrier for *Lactobacillus plantarum* and *Bacillus subtilis* to perform fermentation. The fermentation process preserved the nutrients in the pears and increased the quantity of probiotics. And then we added DPF into the diet of small-tailed Han sheep which aimed to evaluate the benefits of including fermented defective pear in the diet of small-tailed Han sheep to improve their growth, and to assess its impact on their intestinal flora using 16S rRNA analysis. The findings may establish the viability of fermented defective pear as a novel feed additive, offering insights into probiotic usage in meat sheep diets and potentially improving meat sheep productivity.

## 2 Materials and methods

### 2.1 Laboratory animals and design

This experiment was approved by the Animal Ethics Committee of the College of Animal Science and Technology of Tarim University and the China Agricultural University Laboratory Animal Welfare and Animal Experimental Ethical Inspection Committee (No. AW 72303202-1-1).

Sixty male small-tailed Han sheep, all 90 days old and similar body weights (36.03 ± 1.94 kg BW), were selected and divided into 4 groups with three replicates each and five sheep per replicate. During a 7-day adaptation stage, all groups received a basal diet. Subsequently, for 60 days, the sheep were fed different diets: group C (control) received the basal diet, group X the basal diet plus 2% DPF, group Y with 4% DPF, and group Z with 6% DPF.

The experiments were conducted at the Jin Hui Tong sheep farm (Xinjiang, China). The sheep pen was disinfected with 2% NaOH before the experiment began, and sheep were tagged and herded into the pen. The sheep were fed twice daily at 9:30 and 19:30 and had unlimited access to water, with two automatic water fountains in each pen and salt blocks provided.

The feed intake of small tail Han sheep in each treatment group was accurately recorded every day to draw the line chart of feed intake change. The BW, average daily feed intake (ADFI) and feed conversion rate were recorded every 30 days. A total of 3 sheep were sampled from each of the four groups of small-tailed Han sheep on day 60 after 12 h of fasting with the weight of each individual sheep recorded before slaughter.

### 2.2 Defective pear fermentation and diet

DPF was produced from defective pears with added mixed bacteria (*Lactobacillus plantarum* and *Bacillus subtilis*) and fermented for 90 days. The nutritional compositions of DPF are detailed in Table 1, and diets were formulated to meet NRC nutrient requirements, as shown in Table 2.

**Table 1:** The pH, nutritional compositions and antioxidant contents of defective pear fermentation (DPF).

**Ingredient**	**Percentage in the total weight**
pH	4.19
Dry matter	5.03%
Crude protein	3.94%
Neutral detergent fiber	28.33%
Acid detergent fiber	16.52%
Water soluble carbohydrate	17.50%
Total flavonoids	20.58 mg RE/g DW
Total phenolics	16.75 mg RE/g DW

**Table 2:** Ingredients and nutrients of basal diet (dry matter basis).

**Ingredients**	**%**	**Nutritional level**	**%**
Corn	37.48	DE^†^ (MJ/kg)	12.71
DDGS	2.50	ME^†^ (MJ/kg)	10.47
Sunflower kernel cake	1.00	CP^‡^	14.19
Sunflower oil	0.30	Ca^‡^	0.46
Cottonseed meal	14.57	P^‡^	0.30
NaHCO_3_	2.15	NDF^‡^	28.97
NaCl	0.50	ADF^‡^	16.58
Sweet sorghum	20.00		
Corn stalk	20.00		
Premix^*^	1.50		
Total	100.00		

DDGS, Distillers dried grains with solubles; NDF, Neutral detergent fiber; ADF, Acid detergent fiber; CP, Crude protein; DM, Dry matter; DE, Digestible energy; ME, Metabolic energy. ^*^Premix contained (DM basis): Vitamin A (10,000 IU/kg), Vitamin D (5,000 IU/kg), Vitamin E (7.6 IU/kg), copper (9 mg/kg), iron (100 mg/kg), zinc (17 mg/kg), manganese (38 mg/kg), selenium (0.2 mg/kg), calcium (18.58%), phosphorus (1.67%), linoleic acid (0.56%), methionine + cystine (0.16%), lysine (0.14%), threonine (0.14%). ^†^Calculated value. ^‡^Measured value.

### 2.3 Sample collection

Blood samples (~10 mL for each sheep) were collected from the jugular vein at 2 h before slaughtering by using the vacuum blood collection tubes and centrifuged at 3,000 rpm for 15 min and 4°C to obtain the serum sample. These sheep were euthanized and the contents of the rumen, jejunum, and ileum (at day 60) were immediately collected, snap-frozen in liquid nitrogen, and stored at −80°C for analysis of gut microorganisms. The rumen, jejunal and ileal tissues were collected and immediately fixed in 4% paraformaldehyde (Biosharp Co., Ltd., Hefei, China) for subsequent morphological analysis.

### 2.4 Serum analysis

The contents of a group of eight biochemical indices, including superoxide dismutase (SOD), malondialdehyde (MDA), and glutathione peroxidase (GSH-Px), were measured using the kits (Nanjing Jiancheng Bioengineering Institute, Nanjing, China). An automated biochemical analyzer (BK-280, Shandong Blobase Biotechnology Co., Ltd., Shandong, China) was used to measure glucose (GLU), triglycerides (TG), high-density lipoprotein cholesterol (HDL-C), low-density lipoprotein cholesterol (LDL-C), and total cholesterol (TC). All experiments were performed using triplicate biological replicates according to the manufacturer's recommended protocols.

### 2.5 Rumen, jejunum and ileum tissues staining

The pre-fixed samples were cut into small pieces of about 1 square centimeter, then they were covered and cut into thin slices and transferred to a constant temperature water bath at 40°C. After the slices had fully unfolded, let them attach to a glass slide. With 45 min baking at 55–60°C, the slices were haematoxylin-eosin (H&E) strained and measured (Liu et al., 2020). The sections with better morphology were selected and used to observe the morphological characteristics of the rumen, jejunum and ileum by using Image-Pro Plus 6.0 (Media Cybernetics Inc., Bethesda, MD).

### 2.6 DNA extraction and 16S rRNA gene sequencing of rumen, jejunum and ileum contents

The total DNA of microbial community was extracted from the rumen, jejunum and ileum contents, using the E.Z.N.A.^®^ Stool DNA Kit (Omega Bio-tek, Norcross, GA) by following the manufacturer's procedures. The quality of the extracted DNA was estimated with 1% agarose gel electrophoresis. Then this amplification protocol used the universal primers were 338F (5′-ACTCCTACGGGAGGCAGCAG-3′) and 806R (5′-GGACTACHVGGGTWTCTAAT-3′). The genus for key bacterial 16S rRNA in the V3–V4 regions were amplified by the PCR thermocycler (ABI GeneAmR^®^ 9700, Foster City, CA, United States) with the following programs: Denaturation for 3 min at 95°C. Subsequently, a total of 27 cycles were performed, with denaturation for 30 s at 95°C, annealing for 30 s at 55°C, and extension for 45 s at 72°C, ended by the final extension for 10 min at 72°C. The PCR was carried out in a mixture containing 4 μL 5 × TransStart FastPfu buffer, 2 μL 2.5 mM dNTPs, 0.8 μL forward primer (5 μM) and reverse primer (5 μM), 0.4 μL TransStart FastPfu DNA Polymerase, and 10 ng template DNA, with the final volume adjusted to 20 μL using ddH_2_O. The PCR products were collected by 2% agarose gel and purified using the AxyPrep DNA Gel Extraction Kit (Axygen Biosciences, Union City, CA, United States) by following the procedures recommended by the manufacturers. The concentrations of the purified PCR products were determined using the Quantus^TM^ Fluorometer (Promega, United States).

The paired-end sequencing (2 × 300 bp) of the equimolarly pooled purified amplicons was performed on an Illumina MiSeq platform (Illumina, San Diego, CA, United States) using standard procedures recommended by the Majorbio Bio-Pharm Technology Co., Ltd. (Shanghai, China). Paired-end sequence reads were spliced using FLASH version 1.2.11 to generate the splicing sequences, namely raw tags (Magoč and Salzberg, 2011). QIIME version 1.9.1 (Bokulich et al., 2013) has been used for optimization of raw reads. Based on 97% identity with the representative sequences of the OTUs determined and annotated by Uparse version 11, the effective tags were clustered into the operational taxonomic units (OTUs). RDP Classifier version 2.13 based on the 16S rRNA database (Wang et al., 2007) was used to determine the taxonomy of each OTU representative sequence. The alpha diversity indexes (i.e., Chao1, Shannon, and Simpson) were calculated with Mothur version 1.30.2. The Origin software (version 2018, 64 bit) was used for relative abundance analyses at the phylum and genus level. MetaCyc pathways were predicted using PICRUSt 2 version 2.2.0 (Caspi et al., 2020).

### 2.7 Statistical analysis

The significant differences between groups were analyzed by one-way analysis of variance (ANOVA) and Duncan was employed in *post-hoc* tests using the SPSS statistical software version 22.0 (SPSS, Inc., Chicago, IL, United States). Graphs were created using Origin 2018 (OriginLab, Inc., Northampton, MA, United States). The data were shown as the mean ± standard error of the mean (SEM) with the significance levels set at *P* < 0.05 (*) and *P* < 0.01 (**), respectively.

## 3 Results

### 3.1 Effect of DPF on the growth performance of small-tailed Han sheep

The study evaluated the growth performance of small-tailed Han sheep across four groups, examining initial weights, slaughter weights, ADG, ADFI and FCR. The results, detailed in Table 3, indicated no significant differences (*P* > 0.05) in initial weights, slaughter weights and FCR, but there were significant differences in ADG and ADFI (*P* < 0.05) among the groups. The group Y exhibited the highest ADG, showing an 11.03% increase compared to group C. The ranking of average daily feed intake (ADFI) from highest to lowest was: Y, X, C, and Z, with group Y significantly higher (*P* < 0.01) than the other groups. The Supplementary Figure S1 illustrated that group Y maintained the highest ADFI throughout the experiment.

**Table 3:** Effects of defective pear fermentation (DPF) on the growth performance in four groups of small-tailed Han sheep (i.e., groups C, X, Y, and Z).

**Item**	**Group C**	**Group X**	**Group Y**	**Group Z**	***P*-value**
Initial weight (kg)	35.92 ± 0.36	36.20 ± 0.75	35.96 ± 0.56	36.04 ± 0.79	0.99
Slaughter weight (kg)	53.04 ± 0.59	53.81 ± 1.20	54.97 ± 1.26	52.85 ± 1.37	0.54
ADG (g/d)	285.42 ± 6.54^b^	293.64 ± 12.79^b^	316.91 ± 14.20^a^	280.11 ± 12.80^b^	0.02
ADFI (g/d)	1,848.77 ± 40.16^bc^	1,907.63 ± 40.88^b^	2,030.06 ± 42.94^a^	1,791.54 ± 34.64^c^	< 0.01
FCR	6.48 ± 0.13	6.50 ± 0.24	6.41 ± 0.18	6.40 ± 0.26	0.75

The expressions of the data are as mean ± standard error of the mean (SEM) (*n* = 15 sheep for each group). ^a − c^Means with different superscripts within the same row are distinguished. ADG, average daily gain; ADFI, average daily feed intake; FCR, feed conversion rate. Group C = control group; Group X = added with 2% DPF; Group Y = added with 4% DPF; Group Z = added with 6% DPF.

### 3.2 Effect of DPF on the serum characteristics of small-tailed Han sheep

The impact of DPF on the serum characteristics of small-tailed Han sheep is presented in Table 4. Prior to DPF supplementation, there were no significant differences (*P* > 0.05) in the levels of SOD, MDA, and GSH-Px among the four groups. After 60 days of DPF feeding, MDA levels in groups X, Y, and Z were significantly reduced compared to group C (*P* < 0.01), with the most pronounced reduction (46.53%) observed in group Y. Similarly, GSH-Px levels were significantly higher in these groups compared to group C (*P* < 0.01), with group Y showing the highest increase. The levels of GLU, TC, HDL-C, LDL-C, and TG did not significantly differ (*P* > 0.05) among the four groups.

**Table 4:** Effects of defective pear fermentation (DPF) on the serum biochemical indices in the four groups of small-tailed Han sheep (i.e., groups C, X, Y, and Z) in 1 and 60 days.

**Biochemical index**	**Group C**	**Group X**	**Group Y**	**Group Z**	**P-value**
**Day 1**					
SOD (U/mL)	8.69 ± 0.45	9.10 ± 0.74	8.79 ± 0.41	9.15 ± 1.23	0.98
MDA (nmol/mL)	21.11 ± 2.31	21.85 ± 1.56	21.48 ± 1.11	22.11 ± 1.57	0.98
GSH-Px (U/mL)	68.57 ± 1.49	67.10 ± 2.99	69.33 ± 1.82	66.52 ± 1.64	0.76
GLU (mmol/L)	2.46 ± 0.41	2.22 ± 0.40	2.60 ± 0.44	2.48 ± 0.28	0.92
TG (mmol/L)	0.41 ± 0.09	0.34 ± 0.04	0.27 ± 0.05	0.34 ± 0.04	0.50
HDL-C (mmol/L)	1.04 ± 0.04	0.96 ± 0.04	0.91 ± 0.09	0.99 ± 0.06	0.48
LDL-C (mmol/L)	0.62 ± 0.06	0.62 ± 0.05	0.55 ± 0.06	0.51 ± 0.02	0.33
TC (mmol/L)	1.68 ± 0.12	1.64 ± 0.11	1.41 ± 0.06	1.64 ± 0.07	0.25
**Day 60**					
SOD (U/mL)	9.25 ± 0.52	14.19 ± 0.15	15.71 ± 0.62	15.93 ± 0.57	0.21
MDA (nmol/mL)	20.78 ± 2.67^a^	13.04 ± 1.83^c^	11.11 ± 1.31^c^	16.60 ± 0.66^b^	0.02
GSH-Px (U/mL)	86.66 ± 2.10^c^	148.57 ± 8.57^a^	152.92 ± 7.93^a^	107.95 ± 3.63^b^	< 0.01
GLU (mmol/L)	3.00 ± 0.68	3.74 ± 0.14	4.15 ± 0.28	3.98 ± 0.15	0.23
TG (mmol/L)	0.35 ± 0.02	0.28 ± 0.01	0.40 ± 0.02	0.41 ± 0.06	0.10
HDL-C (mmol/L)	0.86 ± 0.01	0.97 ± 0.05	1.14 ± 0.34	0.83 ± 0.02	0.60
LDL-C (mmol/L)	0.43 ± 0.05	0.55 ± 0.03	0.50 ± 0.07	0.56 ± 0.01	0.19
TC (mmol/L)	1.35 ± 0.11	1.47 ± 0.09	1.56 ± 0.11	1.63 ± 0.10	0.31

SOD, Superoxide dismutase; MDA, Malondialdehyde; GSH-Px, Glutathione peroxidase; GLU, Glucose; TG, Triglycerides; HDL-C, High-density lipoprotein cholesterol; LDL-C, Low-density lipoprotein cholesterol; TC, Total cholesterol. The expressions of the data are as mean ± SEM. ^a − c^Means with different superscripts within the same row are distinguished. Group C = control group; Group X = added with 2% DPF; Group Y = added with 4% DPF; Group Z = added with 6% DPF.

### 3.3 Effect of DPF on the intestinal morphology of small-tailed Han sheep

Table 5 presents the effects of DPF on the intestinal morphology of small-tailed Han sheep over 60 days. There were no significant differences observed in the PLs, PWs, and MTs of the rumen among the four groups (*P* > 0.05). However, the rumen PWs tended to widen in the groups supplemented with DPF compared to group C. In the jejunum, VHs and V/C in groups X and Y were significantly increased compared to group C (*P* < 0.01), and CDs in group Y significantly reduced (*P* < 0.05). In the ileum, CDs in the DPF-supplemented groups were significantly shallower than in group C (*P* < 0.05), with group Y having the shallowest CDs. There were no significant differences in VHs and V/C among the four groups (*P* > 0.05).

**Table 5:** Effects of defective pear fermentation (DPF) on the gastrointestinal morphology of the four groups of small-tailed Han sheep (i.e., groups C, X, Y, and Z).

**Location**	**Item**	**Group C**	**Group X**	**Group Y**	**Group Z**	***P*-value**
Rumen (μm)	PL	3,180.48 ± 366.87	3,502.4 ± 310.30	4,352.6 ± 247.20	3,703.22 ± 256.06	0.11
	PW	608.09 ± 94.44	887.40 ± 73.59	1,010.44 ± 93.82	1,043.5 ± 133.36	0.06
	MT	1,064.12 ± 48.00	967.68 ± 92.38	1,135.51 ± 63.84	866.71 ± 74.44	0.12
Jejunum (μm)	VH	397.82 ± 21.17^c^	618.49 ± 42.44^b^	767.41 ± 44.38^a^	364.13 ± 14.68^c^	< 0.01
	CD	237.27 ± 46.33^b^	316.78 ± 25.98^a^	156.06 ± 16.82^c^	211.16 ± 11.82^b^	0.01
	V/C	1.68 ± 0.25^b^	1.95 ± 0.14^b^	4.92 ± 0.65^a^	1.72 ± 0.13^b^	< 0.01
Ileum (μm)	VH	351.23 ± 27.30	460.08 ± 29.92	320.85 ± 26.54	392.17 ± 46.07	0.12
	CD	249.74 ± 23.73^a^	170.21 ± 17.23^c^	153.52 ± 14.63^c^	206.25 ± 11.98^b^	0.02
	V/C	1.47 ± 0.24	2.66 ± 0.32	2.13 ± 0.29	1.97 ± 0.17	0.06

The expressions of the data are as mean ± SEM. ^a − c^Means with different superscripts within the same row are distinguished. PL, papillae length; PW, papillae width; MT, muscular thickness; VH, villus height; CD, crypt depth; V/C, the ratio of VH/CD. Group C = control group; Group X = added with 2% DPF; Group Y = added with 4% DPF; Group Z = added with 6% DPF.

### 3.4 Effect of DPF on the rumen microbiota of small-tailed Han sheep

**Figure 1:**
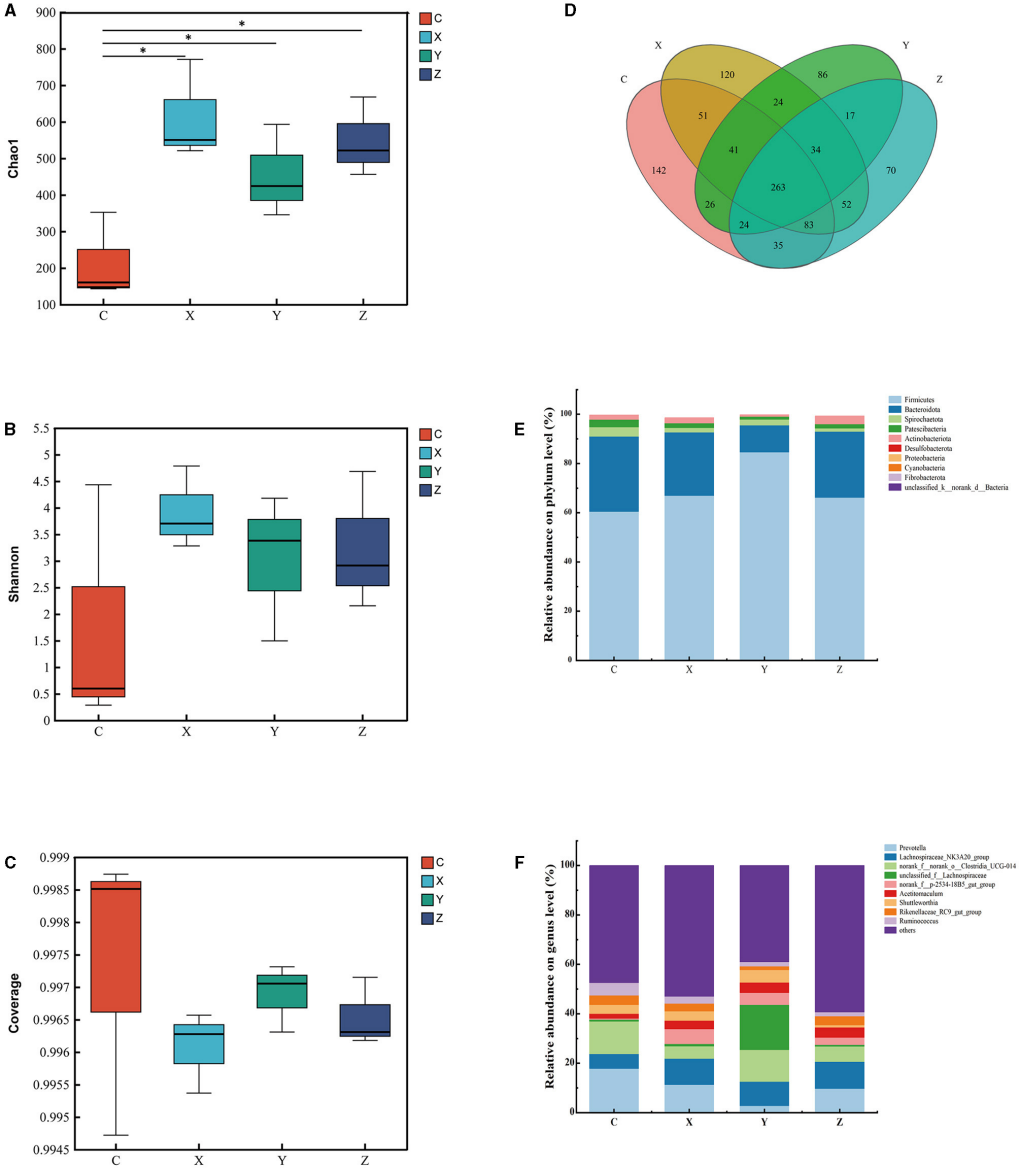
Effect of defective pear fermentation (DPF) on the rumen microbiota in small-tailed Han sheep in 60 days. **(A)** The boxplot of Chao1 index. **(B)** The boxplot of Shannon index. **(C)** The boxplot of coverage index. **(D)** The shared and individual species number on the rumen detected in four groups were showed at Venn diagram in small-tailed Han sheep. **(E)** The relative abundances of the top ten bacterial phyla in the rumen microbiome of small-tailed Han sheep on day 60. **(F)** The relative abundances of the top ten bacterial taxa in the rumen microbiome of small-tailed Han sheep on day 60. Symbol “*” is respectively the expression of the significant differences at *P* < 0.05. Group C = control group; Group X = added with 2% DPF; Group Y = added with 4% DPF; Group Z = added with 6% DPF.

Figure 1 shows the impact of DPF on the rumen microbiota of small-tailed Han sheep after 60 days. Sequencing of the V3–V4 regions of the 16S rRNA gene yielded 263 shared bacterial OTUs, with group C having 142 unique OTUs and group Z 70 in Figure 1D. The Chao1 index, reflecting community richness, was significantly higher in the DPF groups compared to group C (*P* < 0.05; Figure 1A), indicating a substantial increase in rumen community richness following DPF feeding. The Shannon and coverage indexes showed no significant differences among the groups (*P* > 0.05; Figures 1B, C).

At the phylum levels, Table 6 showed that *Firmicutes* and *Bacteroidota* were the most abundant in the rumen microbiota. The relative abundance of *Bacteroidota* decreased as *Firmicutes* increased. In group Y, *Bacteroidota* had a significantly lower abundance than in the other groups (*P* < 0.01; Figure 1E). At the genus level, *Prevotella* was significantly lower in group Y (*P* < 0.01), while *Lachnospiraceae_NK3A20_group* was significantly higher in DPF groups (*P* < 0.05; Figure 1F; Table 6) compared to group C. The abundances of *norank_f_norank_o_Clostridia_UCG-014* were higher in groups C and Y (*P* < 0.05; Figure 1F; Table 6) than in groups X and Z. Group Y showed a notably higher abundance of *unclassified_f_Lachnospiraceae* (*P* < 0.01), and groups X and Y had significantly higher levels of *norank_f_p-2534-18B5_gut_group* (*P* < 0.01; Figure 1F) compared to group C. Besides, there were significantly higher levels of *Acetitomaculum* in groups Y and Z compared to group C.

**Table 6:** Species with different relative abundance of rumen bacteria in different treatments at phylum and genus levels (%).

**Item**	**Group C**	**Group X**	**Group Y**	**Group Z**	***P*-value**
**Phylum**					
*Firmicutes*	60.52 ± 1.93	66.98 ± 10.47	84.63 ± 1.42	66.26 ± 1.40	0.05
*Bacteroidota*	30.57 ± 1.38^a^	25.75 ± 2.24^a^	10.95 ± 0.73^b^	26.83 ± 1.43^a^	< 0.01
**Genus**					
*Prevotella*	17.90 ± 0.76^a^	11.35 ± 0.68^b^	2.83 ± 0.26^c^	9.73 ± 0.63^b^	< 0.01
*Lachnospiraceae_NK3A20_group*	5.91 ± 1.56^b^	10.42 ± 0.93^a^	8.86 ± 1.23^a^	11.00 ± 0.87^a^	0.02
*norank f norank_o Clostridia_UCG-014*	13.18 ± 1.49^a^	5.02 ± 1.77^b^	12.42 ± 1.32^a^	5.80 ± 0.94^b^	0.01
*unclassified_f Lachnospiraceae*	0.77 ± 0.08^b^	0.97 ± 0.03^b^	18.27 ± 0.55^a^	0.68 ± 0.03^b^	< 0.01
*norank_f p-2534-18B5_gut_group*	0.41 ± 0.08^c^	5.95 ± 0.03^a^	4.84 ± 0.55^a^	2.95 ± 0.03^b^	< 0.01
*Acetitomaculum*	1.91 ± 0.02^c^	3.43 ± 0.13^b^	4.15 ± 0.06^a^	4.10 ± 0.06^a^	< 0.01

The expressions of the data are as mean ± SEM. ^a − c^Means with different superscripts within the same row are distinguished. Group C = control group; Group X = added with 2% DPF; Group Y = added with 4% DPF; Group Z = added with 6% DPF.

**Figure 2:**
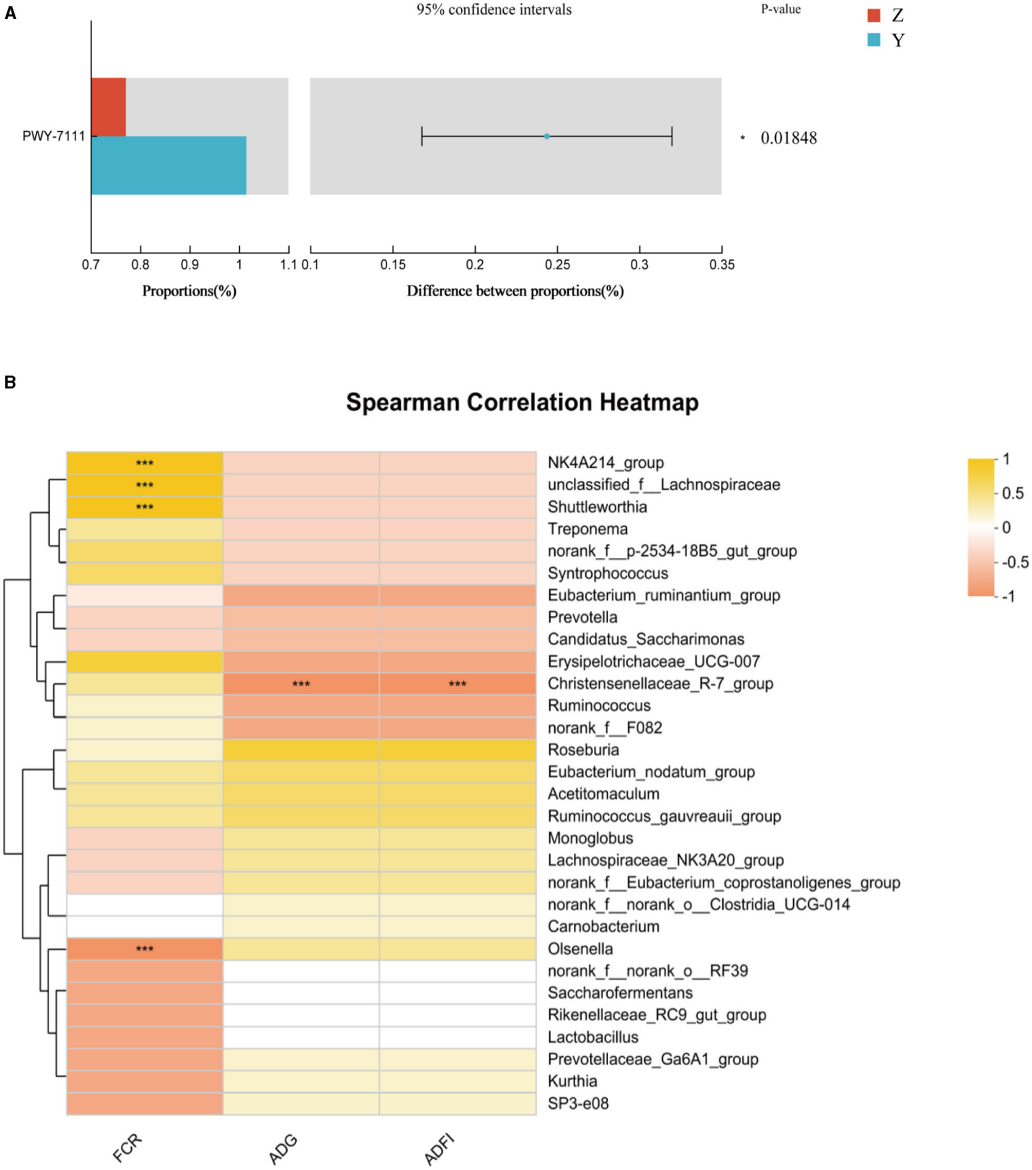
**(A)** Differential MetaCyc pathways of rumen bacteria in different treatment groups of small-tailed Han sheep. **(B)** The bacterial taxa in rumen correlated with growth performance of small-tailed Han sheep. The colors of the squares correspond to correlations: Yellow is positive, but red is negative. Symbol “***” represents a highly significant difference, i.e. *P* < 0.01.

Functional prediction analysis using PICRUSt2 software identified 388 MetaCyc pathways. *T*-tests revealed no significant pathway differences between most groups; however, Pyruvate Fermentation to Isobutanol (PWY-7111) was significantly higher in group Y compared to group Z (*P* < 0.05; Figure 2A).

Correlation analysis at the genus level showed significant associations between specific microbial species and growth performance indicators. The Figure 2B showed that the relative abundances of *NK4A214_group, unclassified_f_Lachnospiraceae*, and *Shuttleworthia* had significant positive correlations (*P* < 0.001) with FCR. *Olsenella* showed a significant negative correlation with FCR (*P* < 0.001), and *Christensenellaceae_R-7_group* had a significant negative correlation (*P* < 0.001) with ADG and ADFI.

### 3.5 Effect of DPF on the jejunum microbiota of small-tailed Han sheep

**Figure 3:**
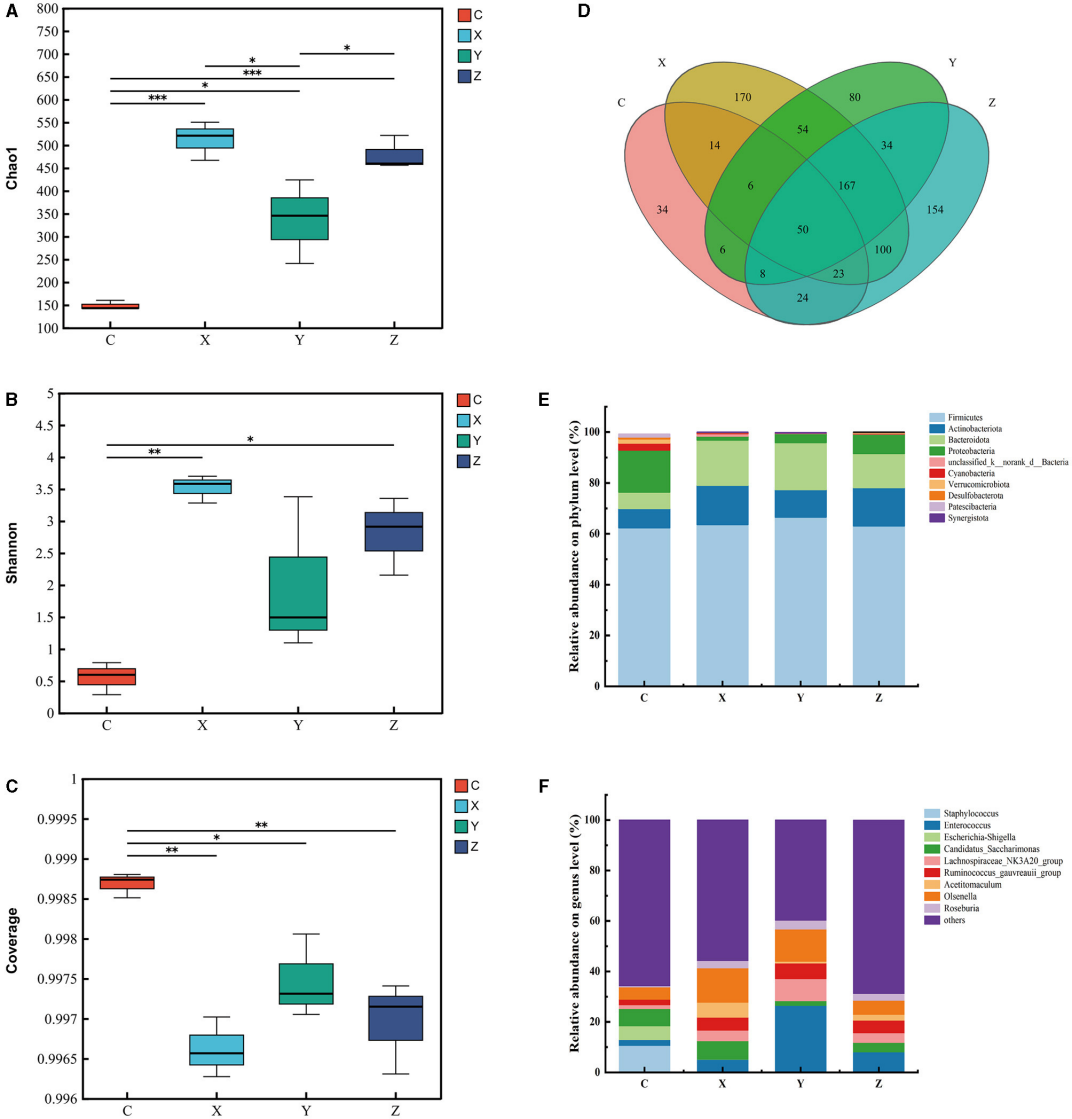
Effect of defective pear fermentation (DPF) on the jejunum microbiota in small-tailed Han sheep in 60 days. **(A)** The boxplot of Chao1 index. **(B)** The boxplot of Shannon index. **(C)** The boxplot of coverage index. **(D)** The shared and individual species number on the jejunum detected in four groups were showed at Venn diagram in small-tailed Han sheep. **(E)** The relative abundances of the top ten bacterial phyla in the jejunum microbiome of small-tailed Han sheep on day 60. **(F)** The relative abundances of the top ten bacterial taxa in the jejunum microbiome of small-tailed Han sheep on day 60. Symbol “*,” “**,” and “***” are respectively the expression of the significant differences at *P* < 0.05, *P* < 0.01 and *P* < 0.001. Group C = control group; Group X = added with 2% DPF; Group Y = added with 4% DPF; Group Z = added with 6% DPF.

Figure 3 illustrates the impact of DPF on the jejunum microbiota of small-tailed Han sheep over a period of 60 days. Sequencing the V3–V4 regions of the 16S rRNA gene in jejunum content samples from the four groups identified 50 shared bacterial OTUs, with 34 unique OTUs in group C and 170 in group X (Figure 3D). The Chao1 index, which indicates community richness, was significantly higher in the DPF-supplemented groups compared to group C (*P* < 0.05; Figure 3A). Notably, groups X and Z had significantly higher Chao1 indices than group Y (*P* < 0.05), with group X also surpassing group Z. The Shannon index, representing community diversity, was significantly higher in groups X and Z compared to group C, while no significant difference was observed between groups Y and C. The coverage index was significantly lower in the DPF groups compared to group C (*P* < 0.05; Figures 3B, C).

Regarding the relative abundances at the phylum level, *Actinobacteriota* and *Bacteroidota* were significantly higher in the DPF groups than in group C (*P* < 0.01; Figure 3E; Table 7) and *Proteobacteria* was significantly lower in the addition groups compared with group C (*P* < 0.05; Figure 3E; Table 7). At the genus level, *Staphylococcus* was significantly more abundant in group C than in the other groups (*P* < 0.01; Figure 3F). Notably, *Enterococcus* and *Howardella* were most abundant in group Y, showing significant differences (*P* < 0.01; Figure 3F; Table 7) compared to the other groups. The relative abundances of *Olsenella* were significantly higher in groups X and Y than in groups C and Z (*P* < 0.01). Group Z had a significantly higher abundance of *Family_XIII_AD3011_group* compared to the other groups.

**Table 7:** Species with different relative abundance of jejunum bacteria in different treatments at phylum and genus levels (%).

**Item**	**Group C**	**Group X**	**Group Y**	**Group Z**	***P*-value**
**Phylum**					
*Actinobacteriota*	7.59 ± 0.50^c^	15.50 ± 0.81^a^	10.85 ± 0.77^b^	15.08 ± 1.11^a^	< 0.01
*Proteobacteria*	16.55 ± 3.72^a^	1.57 ± 0.43^c^	3.64 ± 0.45^c^	7.65 ± 0.21^b^	0.01
*Bacteroidota*	6.45 ± 0.27^c^	17.81 ± 1.00^a^	18.48 ± 0.88^a^	13.33 ± 0.86^b^	< 0.01
**Genus**					
*Staphylococcus*	10.67 ± 1.18^a^	0.01 ± 0.00^b^	0.01 ± 0.01^b^	0.02 ± 0.00^b^	< 0.01
*Enterococcus*	2.33 ± 0.47^c^	5.16 ± 0.27^bc^	26.50 ± 1.94^a^	8.04 ± 1.02^b^	< 0.01
*Olsenella*	4.83 ± 0.40^b^	13.66 ± 1.59^a^	12.76 ± 0.77^a^	5.62 ± 1.27^b^	< 0.01
*Family_XIII_AD3011_group*	0.00 ± 0.00^b^	1.46 ± 0.27^a^	0.33 ± 0.07^b^	2.28 ± 0.47^a^	< 0.01
*Howardella*	0.00 ± 0.00^c^	1.44 ± 0.33^b^	2.45 ± 0.25^a^	0.61 ± 0.17^c^	< 0.01
*Unclassified_f Lachnospiraceae*	0.01 ± 0.00^b^	0.18 ± 0.08^b^	1.25 ± 0.21^a^	0.15 ± 0.01^b^	< 0.01

The expressions of the data are as mean ± SEM. ^a − c^Means with different superscripts within the same row are distinguished. Group C = control group; Group X = added with 2% DPF; Group Y = added with 4% DPF; Group Z = added with 6% DPF.

**Figure 4:**
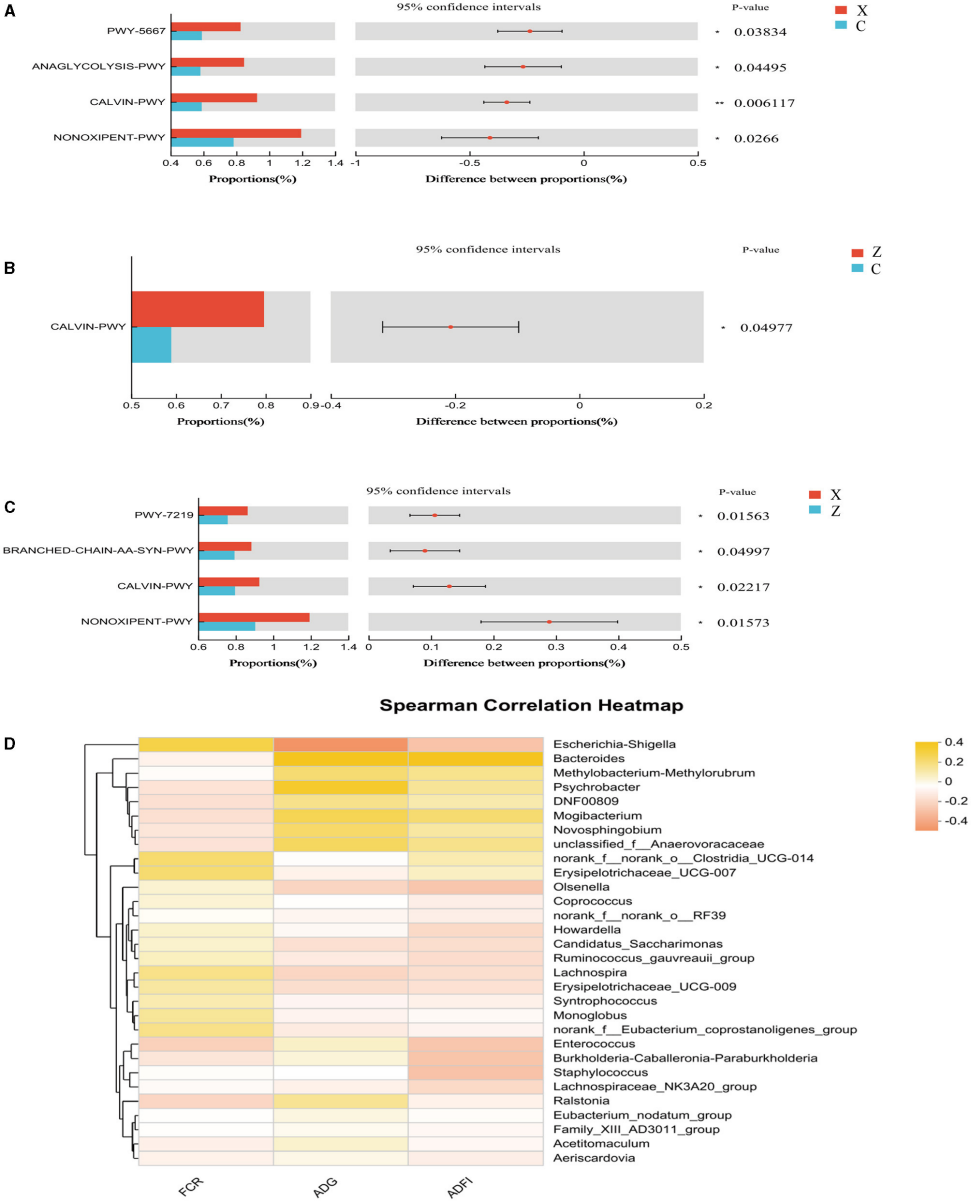
Differential MetaCyc pathways of jejunum bacteria in different treatment groups of small-tailed Han sheep: **(A)** group C and X; **(B)** group C and Z; **(C)** group X and Z. **(D)** The bacterial taxa in jejunum correlated with growth performance of small-tailed Han sheep. The colors of the squares correspond to correlations: Yellow is positive, but red is negative. The significant difference is set at *P* < 0.05 (*), *P* < 0.01 (**), respectively.

Functional prediction analysis using PICRUSt2 software identified 404 MetaCyc pathways. *T-*tests indicated significant differences (*P* < 0.05; Figure 4A) in four pathways between groups C and X: CDP-diacylglycerol biosynthesis I (PWY-5667), glycolysis III (ANAGLYCOLYSIS-PWY), the Calvin-Benson-Bassham cycle (CALVIN-PWY), and the non-oxidative branch of the pentose phosphate pathway (NONOXIPENT-PWY), with higher proportions in group X. Group Z showed significantly higher proportions of CALVIN-PWY compared to group C (*P* < 0.05; Figure 4B). Between groups X and Z, four pathways showed significant differences (*P* < 0.05; Figure 4C): Adenosine ribonucleotides de novo biosynthesis (PWY-7219), Superpathway of branched amino acid biosynthesis (BRANCHED-CHAIN-AA-SYN-PWY), CALVIN-PWY, and NONOXIPENT-PWY, with higher proportions in group X.

Spearman correlation analysis revealed no significant correlations between species at the genus level in the jejunum microbiota and growth performance indicators in small-tailed Han sheep (Figure 4D).

### 3.6 Effect of DPF on the ileum microbiota of small-tailed Han sheep

**Figure 5:**
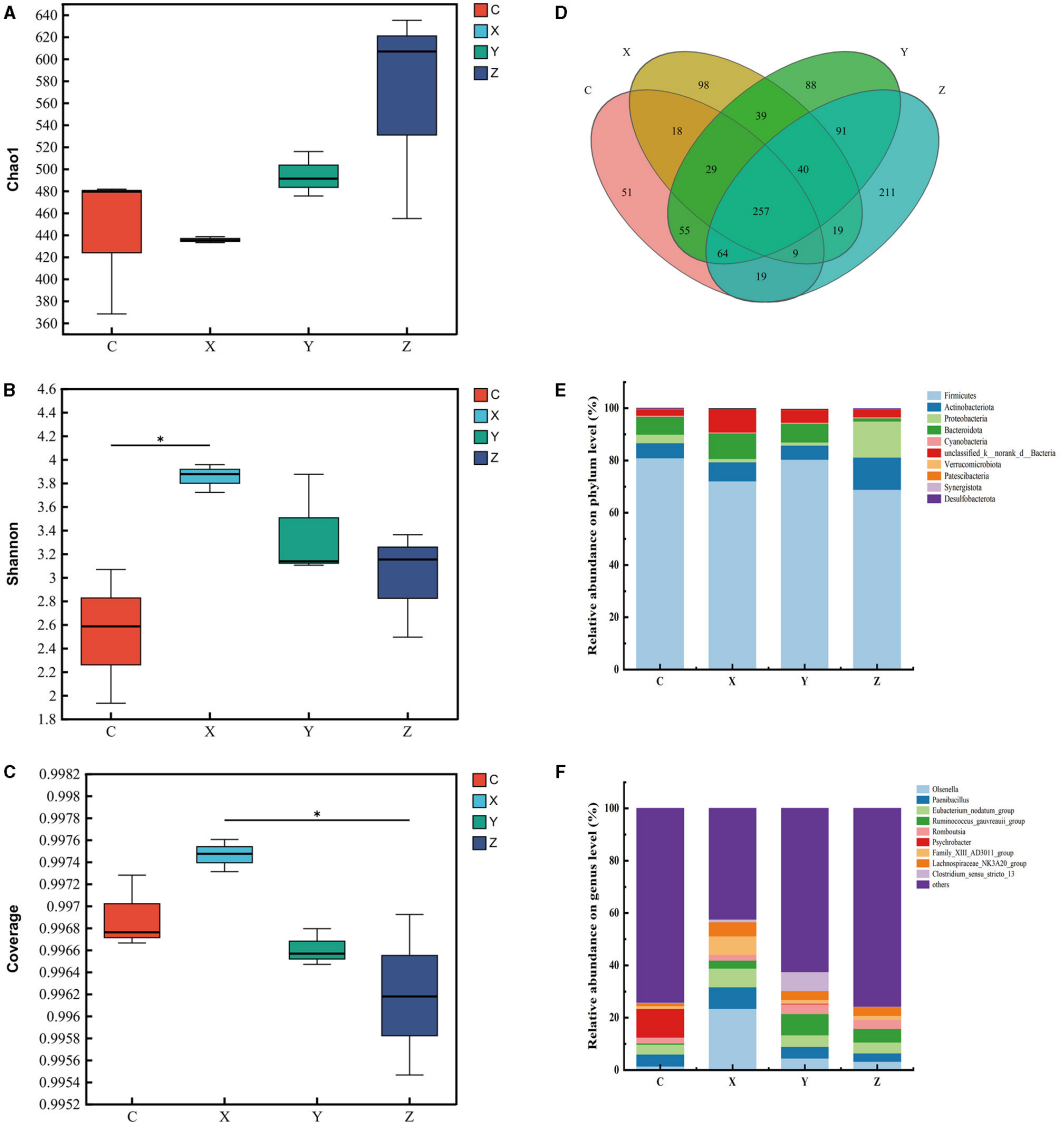
Effect of defective pear fermentation (DPF) on the ileum microbiota in small-tailed Han sheep in 60 days. **(A)** The boxplot of Chao1 index. **(B)** The boxplot of Shannon index. **(C)** The boxplot of coverage index. **(D)** The shared and individual species number on the ileum detected in four groups were showed at Venn diagram in small-tailed Han sheep. **(E)** The relative abundances of the top ten bacterial phyla in the ileum microbiome of small-tailed Han sheep on day 60. **(F)** The relative abundances of the top ten bacterial taxa in the ileum microbiome of small-tailed Han sheep on day 60. Symbol “*” is respectively the expression of the significant differences at *P* < 0.05. Group C = control group; Group X = added with 2% DPF; Group Y = added with 4% DPF; Group Z = added with 6% DPF.

Figure 5 displays the effects of DPF on the ileum microbiota of small-tailed Han sheep over 60 days. Sequencing the V3–V4 regions of the 16S rRNA gene in ileum content samples from the four groups identified 257 shared bacterial OTUs, with group C having 51 unique OTUs and group Z 211 (Figure 5D). The Chao1 index, indicating community richness, showed no significant differences among the treatment groups (*P* > 0.05; Figure 5A). However, group X exhibited a significantly higher Shannon index (reflecting community diversity) compared to group C (*P* < 0.05; Figure 5B), and its Coverage index was also significantly higher than that of group Z (*P* < 0.05; Figure 5C).

The analysis revealed that the relative abundances of *Firmicutes* were significantly higher in groups C and Y than in groups X and Z (*P* < 0.05; Figure 5E; Table 8). *Actinobacteriota* was significantly less abundant in groups X, Y, and Z compared to group Z (*P* < 0.05), while group X had the highest relative abundance of *Bacteroidota* (*P* < 0.01; Figure 5E; Table 8). At the genus level, *Psychrobacter* was significantly less abundant in the DPF-supplemented groups than in group C (*P* < 0.01; Figure 5F; Table 8). Additionally, the relative abundances of *Olsenella, Howardella, DNF00809*, and *Monoglobus* were significantly higher in group X compared to the other groups (*P* < 0.01; Figure 5F; Table 8). Group Y had the highest relative abundances of *Romboutsia, Ruminococcus_gauvreauii_group*, and *Clostridium_sensu_stricto_13* among four groups (*P* < 0.01; Figure 5F; Table 8).

**Table 8:** Species with different relative abundance of ileum bacteria in different treatments at phylum and genus levels (%).

**Item**	**Group C**	**Group X**	**Group Y**	**Group Z**	***P*-value**
**Phylum**					
*Firmicutes*	81.14 ± 2.38^a^	71.32 ± 0.50^b^	80.78 ± 3.14^a^	67.68 ± 3.87^b^	0.02
*Actinobacteriota*	5.37 ± 1.27^b^	7.24 ± 1.12^b^	5.23 ± 0.99^b^	11.14 ± 0.58^a^	0.01
*Bacteroidota*	6.68 ± 0.38^b^	9.79 ± 0.24^a^	7.18 ± 0.11^b^	1.20 ± 0.21^c^	< 0.01
**Genus**					
*Psychrobacter*	10.96 ± 1.38^a^	0.00 ± 0.00^b^	0.35 ± 0.04^b^	0.05 ± 0.02^b^	< 0.01
*Olsenella*	1.50 ± 0.32^b^	23.49 ± 1.99^a^	4.52 ± 0.24^b^	3.31 ± 0.71^b^	< 0.01
*Clostridium_sensu_stricto_13*	0.14 ± 0.04^c^	1.04 ± 0.28^b^	7.20 ± 1.27^a^	0.11 ± 0.03^c^	< 0.01
*Ruminococcus_gauvreauii_group*	0.51 ± 0.15^c^	3.01 ± 0.93^b^	8.12 ± 1.85^a^	5.17 ± 1.32^b^	< 0.01
*Howardella*	0.13 ± 0.04^b^	4.54 ± 0.44^a^	0.65 ± 0.04^b^	0.50 ± 0.02^b^	< 0.01
*Romboutsia*	2.20 ± 0.10^c^	2.16 ± 0.37^c^	5.69 ± 0.13^a^	3.59 ± 0.37^b^	< 0.01
*DNF00809*	0.27 ± 0.04^c^	2.54 ± 0.33^a^	0.38 ± 0.02^c^	0.96 ± 0.08^b^	< 0.01
*Monoglobus*	0.46 ± 0.02^c^	1.97 ± 0.25^a^	1.41 ± 0.07^b^	0.22 ± 0.03^c^	< 0.01

The expressions of the data are as mean ± SEM. ^a − c^Means with different superscripts within the same row are distinguished. Group C = control group; Group X = added with 2% DPF; Group Y = added with 4% DPF; Group Z = added with 6% DPF.

**Figure 6:**
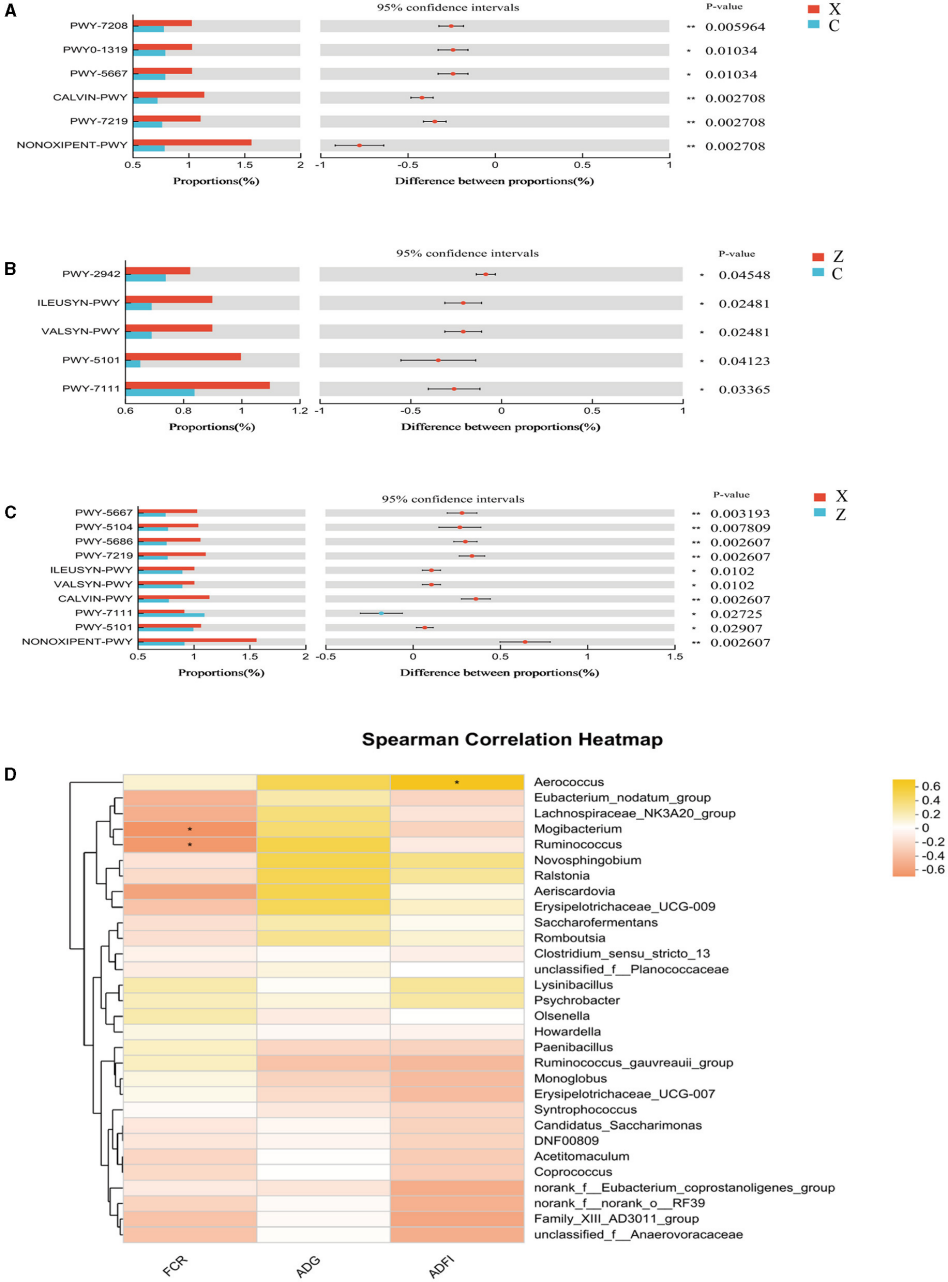
Differential MetaCyc pathways of ileum bacteria in different treatment groups of small-tailed Han sheep: **(A)** group C and X; **(B)** group C and Z; **(C)** group X and Z. **(D)** The bacterial taxa in ileum correlated with growth performance of small-tailed Han sheep. The colors of the squares correspond to correlations: Yellow is positive, but red is negative. Symbol “^*^” represents a significant difference, i.e. *P* < 0.05.

Functional prediction analysis using PICRUSt2 software identified 403 MetaCyc pathways. *T*-tests indicated significant differences in six pathways between groups C and X: Superpathway of pyrimidine nucleobases salvage (PWY-7208), CDP-diacylglycerol biosynthesis II (PWY0-1319), CDP-diacylglycerol biosynthesis I (PWY-5667), CALVIN-PWY, Adenosine ribonucleotides *de novo* biosynthesis (PWY-7219), and NONOXIPENT-PWY, with group X showing higher proportions (Figure 6A). Group Z had significantly higher proportions in five pathways compared to group C: L-lysine biosynthesis III (PWY-2942), L-isoleucine biosynthesis I (ILEUSYN-PWY), L-valine biosynthesis (VALSYN-PWY), L-isoleucine biosynthesis II (PWY-5101), and PWY-7111 (Figure 6B). Ten pathways were significantly more abundant in group X compared to group Z: PWY-5667, L-isoleucine biosynthesis IV (PWY-5104), UMP biosynthesis (PWY-5686), PWY-7219, ILEUSYN-PWY, VALSYN-PWY, CALVIN-PWY, PWY-7111, PWY-5101, and NONOXIPENT-PWY (Figure 6C).

Spearman correlation analysis found significant associations between certain genus-level species in the ileal microbiota and growth performance indicators. Figure 6D revealed that the abundance of *Aerococcus* (*P* < 0.05) was positively correlated with ADFI, while *Mogibacterium* and *Ruminococcus* showed negative correlations with FCR (*P* < 0.05).

## 4 Discussion

### 4.1 Effects of DPF on the growth performance of small-tailed Han sheep

The nutritional value of feed is primarily determined by its chemical composition, but its effectiveness depends on how it's digested and metabolized by animals, thereby fulfilling their physiological needs (Gao, 2023). In this study, the inclusion of group Y's diet in small-tailed Han sheep led to a significant increase in ADFI compared to group C. This suggested that the fermentation process, which included specific probiotics and a distinctive fruit flavor (Guo, 2022), may have enhanced the palatability of the total mixed ration (TMR). Similar findings, such as the unique probiotic fermentation aroma of Brewers' spent grain improving feed intake and weight gain in lambs, support this observation (Feksa Frasson et al., 2018). Group Z showed the lowest ADFI, likely due to an excessive amount of defective pear fermentation and high sugar content, increasing the TMR's viscosity and altering its texture, thus reducing feed intake.

ADG increased with the increase of ADFI, and there was a positive correlation between them (Foote et al., 2017; Saleem et al., 2017; Flohr et al., 2018; Devyatkin et al., 2021; Zhou et al., 2023). However, the increase of ADFI does not necessarily increase FCR. For example, as reported by Ferronato et al. (2024), the feed intake of broilers in the grower period increased significantly after vitamin C and acetylsalicylic acid were added, but there was no significant change in FCR. The influence of feed pH on feed intake is mainly due to the acidity impacting the palatability of the feed itself. For example, Wang (2022) reported that when excessive fermented pomegranate peel was added to the feed of broilers, high tannic acid content made the feed itself sour and palatability decreased, resulting in lower feed intake and weight gains in the treated group. In this study, although ADG and ADFI in groups X and Y were significantly increased, slaughter weight was not affected. The reason for this phenomenon may be that the pH of TMR decreased slightly after the addition of DPF, while the internal environment of the rumen was in a relatively stable neutral environment for a long time, and the decrease of feed pH may have a certain impact on rumen digestion and absorption, which resulted in no significant difference in slaughter weight and FCR.

### 4.2 Effects of DPF on the serum characteristics in small-tailed Han sheep

Pears, as one of the most well-known fruits around the world, contain a certain amount of flavonoids and phenolic compounds in their flesh, skin, and core, which are known to have antioxidant properties (Teixeira et al., 2023). However, an animal's antioxidant defense system is integral to its health. The primary roles of GSH-Px and SOD are to eliminate free radicals (Song and Shen, 2020; Zheng et al., 2023). Malondialdehyde, a byproduct of lipid peroxidation in cells, can damage cell membrane structure and function (Meng et al., 2023). In this study, the defective pears still retained a certain amount of flavonoids and phenolic compounds after they were made into fermentation (Table 1). So the antioxidant capacity of sheep in the groups with added DPF were better than that in the group without added DPF.

GLU is an energy source crucial for the growth of small-tailed Han sheep, reflecting their energy metabolism state (Graugnard et al., 2012). TC, HDL-C, LDL-C, and TG are important lipid markers (Agongo et al., 2022). Since the sheep in this study were raised in captivity, these indicators were monitored to ensure normal growth without excessive fat accumulation. The results indicated no abnormal fat accumulation in any group.

### 4.3 Effects of DPF on the gastrointestinal morphology of small-tailed Han sheep

The rumen of ruminants is a complex ecosystem vital for the initial digestion and absorption of nutrients such as protein, starch, and fiber in feed (Norouzian et al., 2011; Zeitz et al., 2016). Key structural aspects of the rumen, such as papilla height (PL), width, and muscle layer thickness (MT), influence its digestive and absorptive functions. These structures enhance feed nutrient digestion and absorption by increasing the surface area in contact with feed (Górka et al., 2018; Zhang K. et al., 2021). In this study, while there were no significant differences in various indicators, group Y exhibited the highest levels of PLs and MTs. A higher PL increases the contact area with ingested feed, and a thicker MT indicates stronger rumen peristalsis ability in group Y, leading to improved rumen digestion and absorption. This correlates with the observed changes in ADG and ADFI in group Y, which were the highest among the four groups, due to the better development of ruminal PLs and MTs.

Typically, an animal's gut is divided into anterior segment and posterior segments. The anterior segment, including the jejunum and ileum, is the primary site for digestion and absorption. Feed initially digested in the rumen turns into chyme, which is mainly digested and absorbed in the jejunum and ileum (Wang et al., 2020). Villus height (VH), crypt depth (CD), and the villus-to-crypt ratio (V/C) are critical indicators of the small intestine's health and the digestive and absorptive capacity of sheep (Han et al., 2022). VH determines the contact area between the intestinal lining and chyme and is positively correlated with this area (Awad et al., 2011). As CD increases, villi shrink, reducing digestion. V/C ratio is an indicator of small intestine function (Yu et al., 2022).

In this study, group Y showed the highest VH and V/C ratios and the lowest CD in the jejunum, indicating superior intestinal health and digestive and absorptive capabilities compared to other groups. Group X's VH was second only to group Y, but its CD was the highest, leading to a V/C ratio comparable to groups C and Z. Although the VH in group Z's jejunum was not significantly different from group C, it was the lowest among all groups. Thus, excessive DPF addition (6%) may negatively impact intestinal health and function.

The CD of the ileum in each treatment group merits attention. As DPF amounts increased, CD initially decreased then increased, particularly at a 6% supplementation level. These structural changes suggest that the DPF amount should be optimal. This also explains the poor growth performance of group Z, likely due to diminished intestinal health and reduced digestive and absorptive functions.

### 4.4 Effects of DPF on the rumen microbiota in small-tailed Han sheep

The rumen, a crucial digestive organ in ruminants, significantly impacts their digestive system, particularly through its microbial structure. Understanding the relationship between rumen microbiota and growth performance is essential for enhancing the growth of small-tailed Han sheep. In this study, we compared and explored the bacterial communities in rumen content samples across four treatment groups. The results indicated a significant increase in species richness of bacterial communities with the addition of DPF, although the Shannon and coverage indices showed no significant differences. At the phylum level, the microbial composition of the rumen is relatively stable, predominantly comprising *Firmicutes, Bacteroidota, Fibrobacteres*, and *Proteobacteria* (McCann et al., 2014; Deusch et al., 2017; Ge et al., 2023). In line with previous studies, the rumen microbiota in our study was dominated by *Firmicutes* and *Bacteroidota*, collectively accounting for over 90% of the total abundance in each treatment group. Common dominant genera included *unidentified Prevotellaceae, Fibrobacter, unidentified Lachnospiraceae, Saccharofermentans*, and *Succinivibrio* (Dai et al., 2015; Zhang Y. K. et al., 2021). Our findings (Figure 1F; Table 6) mirrored these trends, showing high relative abundances of *Prevotella, Lachnospiraceae_NK3A20_group, norank_f_norank_o_Clostridia_UCG-014*, and *unclassified_f_Lachnospiraceae* at the genus level. Notably, the relative abundance of *Prevotella* was significantly lower in the DPF-supplemented groups compared to group C. This could be due to the inhibition of *Prevotella* growth by higher abundances of *Lachnospiraceae_NK3A20_group* in the supplemented groups. Specifically, group Y had the highest abundance of *unclassified_f_Lachnospiraceae* but the lowest of *Prevotella*, suggesting a possible antagonistic relationship between *Prevotella* and *Lachnospiraceae*. *unclassified_f_Lachnospiraceae*, a potential probiotic, played a role in the metabolism of various carbohydrates and the fermentation of intermediate lactate and acetate to butyrate, aiding in maintaining rumen homeostasis and feed digestion (Paz et al., 2018; Zhang et al., 2022). This aligns with our findings, which revealed a significant positive correlation between *unclassified_f_Lachnospiraceae* and FCR, but slightly different, the increase of ADG and ADFI in group Y did not have significant effects on slaughter weight and FCR. Therefore, we can only assume that DPF has the potential to improve the growth performance of small-tailed Han sheep. Additionally, bacterial function prediction indicated that Pyruvate Fermentation to Isobutanol pathway was enriched in group Y (Figure 2A). Given that isobutanol production requires one NADPH molecule per molecule, higher isobutanol levels can enhance animals' antioxidant capacity (Sauer and Eikmanns, 2005). Consequently, the antioxidant capacity of sheep in group Y was stronger than that in group Z.

### 4.5 Effects of DPF on the jejunum microbiota in small-tailed Han sheep

The jejunum is a critical site for nutrient absorption in animals, and a healthy jejunal environment is essential for their growth and development. Our analysis of alpha diversity indicated a significant increase in species richness in the groups supplemented with DPF. Microbial community diversity was notably higher in groups X and Z compared to groups Y and C (Figure 3B). The coverage for each group exceeded 0.99, ensuring the reliability of the detection results (Figure 3C). At the phylum level, an important observation (Figure 3E; Table 7) was made regarding the relative abundances of bacteria in the jejunum. Group C exhibited the highest relative abundance of Proteobacteria but the lowest of *Bacteroidota* among all groups (Pitta et al., 2014). Previous research suggests that *Bacteroidota* are more efficient in degrading carbohydrates and polysaccharides than *Proteobacteria*, which could explain the reduced digestion and absorption capabilities, and consequently poorer growth performance, in group C. Additionally, the presence of *Staphylococcus*, of which more than half are pathogenic, was noted in group C (Hindieh et al., 2022). In contrast, only minimal amounts of *Staphylococcus* were detected in the DPF-supplemented groups, indicating that DPF addition to the diet of small-tailed Han sheep could effectively reduce *Staphylococcus* levels in the jejunum (Figure 3F; Table 7). Moreover, group Y had the highest relative abundance of *Enterococcus* (Figure 3F; Table 7), a potential probiotic known to withstand digestive stress and benefit host health, possibly contributing to the superior growth performance of sheep in this group (Siddique et al., 2021). Bacterial function prediction revealed that most metabolic pathways were enriched in group X (Figures 4A–C), with significant differences from groups C and Z. For instance, the NONOXIPENT-PWY pathway, closely associated with glycolysis in the body, suggests that stronger glycolysis capability, as seen in group X, yields more energy conducive to bodily activities. Hence, the proportion of NONOXIPENT-PWY in group X was slightly higher than in groups C and Z (Figures 4A–C).

### 4.6 Effects of DPF on the ileum microbiota in small-tailed Han sheep

The ileum, situated after the jejunum, plays a crucial role in digestion and absorption. Alpha diversity analysis showed that group X had the highest Shannon index, indicating a more diverse bacterial community in this group (Figure 5B). The bar chart representing genus-level relative abundance revealed that group C had the highest abundance of *Psychrobacter* (Figure 5F). Some research suggests that *Psychrobacter* may be a potential probiotic positively affecting animal growth (Yang et al., 2011; Makled et al., 2017). However, *Psychrobacter* was not detected in the groups supplemented with DPF in this study, likely due to the inhibitory effect of other bacteria in these groups. Notably, group Y had the highest abundance of the *Ruminococcus_gauvreauii_group* (Figure 5F; Table 8), crucial in starch degradation (Flint et al., 2008; Chassard et al., 2012). Group Y exhibited the highest abundance (Figure 5F; Table 8) of *Clostridium_sensu_stricto_13*, a member of the *Clostridium* genus and a symbiotic bacterium in the animal gut known for producing spores that withstand environmental stress, thus aiding gut health (Guo et al., 2020). There is varied research regarding *Olsenella*. Some studies identify it as an anaerobic bacterium that produces lactic acid from glucose fermentation, similar to *Lactobacillus*, and is considered a probiotic aiding feed digestion (Gaowa et al., 2021). However, McLoughlin et al. (2020) found a negative correlation between *Olsenella* and FCR. Our study differs from this finding, *Mogibacterium* and *Ruminococcus* were negatively correlated with FCR (Figure 6D). However, given the growth performance of DPF2, *Olsenella* appeared to positively influence the growth and development of sheep in our study, tentatively categorizing it as a potential probiotic. Like in the jejunum, group X had more enriched MetaCyc pathways, with no significant differences in pathway proportions between groups X and Y (Figures 6A–C).

## 5 Conclusion

In conclusion, our study demonstrated the positive effects of using 2% to 4% DPF as feed additives on the growth performance of small-tailed Han sheep. This supplementation notably increased both the ADG and ADFI of these sheep. Furthermore, incorporating DPF into the diet significantly enhanced the antioxidant capacity of the small-tailed Han sheep. The gastrointestinal microbiota compositions were notably altered by DPF addition, primarily elevating the relative abundance of *Firmicutes* and suppressing Gram-negative bacteria, including *Prevotella*. The addition of 4% DPF notably increased the relative abundance of *unclassified_f_Lachnospiraceae* in the rumen. Concurrently, there was a marked increase in the relative abundance of *Bacteroidota* and a decrease in *Proteobacteria*, with a significant increase in *Enterococcus*, especially in group Y within the jejunum. Additionally, the relative abundances of *Olsenella* and *Ruminococcus_gauvreauii_group* in jejunum segment and ileum segment were increased after the addition of DPF. At the same time, an increase of the relative abundance of *Olsenella* in group X in the ileum segment was particularly prominent. Bacterial function prediction indicated that energy metabolism-related pathways, such as NONOXIPENT-PWY, were more prevalent in groups X and Y, potentially boosting the sheep's metabolic capacity. These findings preliminarily confirm the viability of using DPF as a feed additive, suggesting a 2% to 4% inclusion rate in the diet of small-tailed Han sheep. However, further research is needed to explore the broader implications of DPF use in animal production, particularly in regions lacking high-quality feed.

## Data availability statement

The datasets presented in this study can be found in online repositories. The names of the repository/repositories and accession number(s) can be found below: https://www.ncbi.nlm.nih.gov/, PRJNA1050385.

## Ethics statement

The animal studies were approved by the Animal Ethics Committee of the College of Animal Science and Technology of Tarim University and the China Agricultural University Laboratory Animal Welfare and Animal Experimental Ethical Inspection Committee (No. AW 72303202-1-1). Tarim University is my affiliation, and China Agricultural University is the cooperative institution of the project. The studies were conducted in accordance with the local legislation and institutional requirements. Written informed consent was obtained from the owners for the participation of their animals in this study.

## Author contributions

HP: Conceptualization, Data curation, Formal analysis, Investigation, Methodology, Software, Writing – original draft, Writing – review & editing. PC: Methodology, Writing – review & editing. RG: Methodology, Writing – review & editing. ZZ: Methodology, Writing – review & editing. YC: Methodology, Writing – review & editing. PX: Methodology, Writing – review & editing. HS: Conceptualization, Resources, Writing – review & editing. YX: Writing – review & editing. HJ: Conceptualization, Resources, Writing – review & editing.

## Supplementary material

Supplementary Figure S1 Effect of defective pear fermentation (DPF) on average daily feed intake of small-tailed Han sheep in 60 days. Group C = control group; Group X = added with 2% DPF; Group Y = added with 4% DPF; Group Z = added with 6% DPF.
