# Supplementary figures and images for: Regulation of the growth performance and the gastrointestinal microbiota community by the addition of defective pear fermentation to feed of small-tailed Han sheep
Source: Front Microbiol. 2024 Apr 4;15:1358033. doi: 10.3389/fmicb.2024.1358033 (PMC11024308; doi:10.3389/fmicb.2024.1358033)

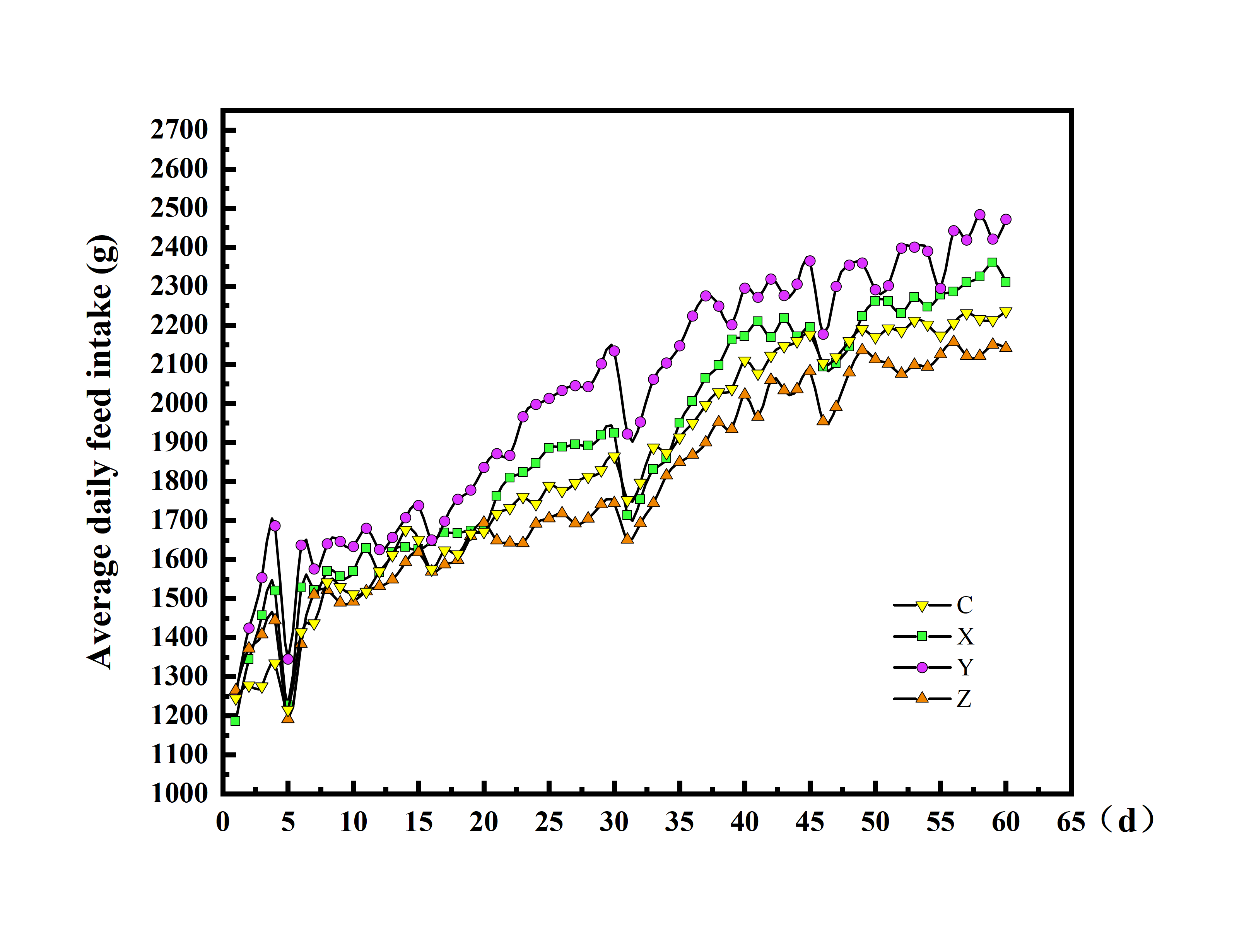

Supplement: Supplementary Figure S1 — Effect of defective pear fermentation (DPF) on average daily feed intake of small-tailed Han sheep in 60 days. Group C = control group; Group X = added with 2% DPF; Group Y = added with 4% DPF; Group Z = added with 6% DPF. [file Image_1.TIF]
